# Leukocytospermia in late adolescents: possible clinical interpretations

**DOI:** 10.1007/s40618-020-01462-8

**Published:** 2020-11-23

**Authors:** S. La Vignera, R. Cannarella, A. Aversa, R. Rago, R. A. Condorelli, A. E. Calogero

**Affiliations:** 1 Department of Clinical and Experimental Medicine, University of Catania, Policlinico “G. Rodolico”, via S. Sofia 78, 95123 Catania, Italy; 2 Department of Experimental and Clinical Medicine, “Magna Graecia” University, 88100 Catanzaro, Italy; 3 Physiopathology of Reproduction and Andrology Unit, Sandro Pertini Hospital, 00157 Rome, Italy

**Keywords:** Testicular volume, Male infertility, Primary prevention, Leukocytospermia, Immature germ cells, Oligozoospermia

## Abstract

**Background:**

No data are currently available on the implication of amicrobial leukocytospermia in male adolescents. Therefore, the primary aim of this study was to evaluate the prevalence of amicrobial leukocytospermia among non-smoker late adolescents who were exposed to other risky lifestyles for the andrological health. The main andrological clinical features of adolescents with leukocytospermia were also reported.

**Methods:**

This is a cross-sectional study carried out in 80 boys. Each adolescent underwent a physical examination, and to the assessment of sperm conventional parameters, seminal leukocytes concentration and immature germ cell evaluation. A possible correlation between seminal leukocytes and immature germ cells and testicular volume (TV) was tested.

**Results:**

The adolescents enrolled in this study had 18.0 ± 0.4 (range 18.1–18.9) years. Unprotected sexual intercourse was referred by 38% of them. Sexual dysfunctions were found in 25% and isolated hypoactive sexual desire in 12.5% of boys. Low TV and penile length in flaccidity were found in 44% and 30% of them, respectively. Only 41% had normozoospermia at the sperm analysis, whereas 19% had isolated oligozoospermia, 15% oligo-asthenozoospermia, and 25% oligo-astheno-teratozoospermia. Leukocytospermia occurred in 25% (20 out of 80) of adolescents. No seminal infection was detected in 19% (15 out of 80) of them. Adolescents with leukocytospermia had lower progressive sperm motility, percentage of normal forms, TV, and a higher percentage of immature germ cells compared to those without leukocytospermia. Semen leukocyte concentration correlated negatively with TV and positively with the percentage of immature germ cells in the ejaculate.

**Conclusion:**

Leukocytospermia, increased immature germ cell number, and low TV identify a distinct phenotype suggestive of testicular tubulopathy. Primary prevention of male infertility and the counselling for andrological risky lifestyles is mandatory and should be started as early as possible.

## Introduction

There is much debate about when the first sperm analysis should be requested, although evidence suggests no earlier than a year and a half after the onset of puberty [1]. Conventionally, the steps preceding full sexual maturation include spermaturia (the presence of spermatozoa in the urine) and ejacularche (the first conscious ejaculation) [1]. In addition to the sperm analysis, a testicular volume (TV) lower than that normally expected for the Tanner stage, the maintenance of high serum levels of anti-Müllerian hormone (AMH), and low inhibin B values may be additional useful criteria to carefully monitor the transitional age during puberty [2–4].

Sperm analysis should be performed by a fully-trained and experienced seminologist engaged in a quality control program and interpreted by a trained clinician. The presence of abnormal conventional sperm parameters (concentration, motility, and morphology) results in a diagnosis of oligozoospermia (sperm count < 15 × 10^6^/ml and total sperm count < 39 million/ejaculate), asthenozoospermia (progressive sperm motility < 32% and total motility < 40%), and/or teratozoospermia (normal forms < 4%) [5]. A second sperm analysis is, however, mandatory because of the elevated intra-individual variations also in men with normal TV and/or normal serum levels of gonadotropins, inhibin B and AMH.

The association between varicocele and abnormal sperm parameters is well known in the adulthood [6]. By contrast, few evidence is available on this matter in the adolescence [3], when testicular asymmetry, scrotal pain, and/or hormone abnormalities suggest the correction of varicocele [7, 8]. Several other diseases are able to affect sperm parameters, such as genetic disorders (karyotype abnormalities, numeric or structural chromosome alterations, Y chromosome microdeletions, syndromic genetic disorders, single gene mutations) [9, 10], male accessory gland infection or inflammation [11], congenital or acquired hypogonadism, testicular tumor, testicular torsion, chemotherapy, and radiotherapy. Importantly, pre-natal exposure to endocrine disruptors or maternal cigarette smoke can similarly damage spermatogenesis in the adulthood [12]. Furthermore, cigarette smoke, marijuana, alcohol intake, obesity during adolescence can also negatively impact on spermatogenesis [13–15].

The finding of an increase in the concentration of leukocytes in the semen in adolescents is still matter of debate. However, the close relationship between leukocytospermia (> 1 million/ml) and urogenital infections is widely accepted and, therefore, the microbiological assessment is currently suggested in these patients [17]. A low leukocyte concentration, by causing a limited sperm exposure to reactive oxygen species, associates with a better sperm quality [18–20]. Based on these premises, the study aims were twofold. First, to evaluate the prevalence of leukocytospermia in non-smoker, healthy (with no major andrological or general comorbidities) late adolescents, enrolled in an andrology screening program carried out in the city of Catania (Italy). The second purpose was to assess the main andrological clinical characteristics of adolescents with leukocytospermia.

## Patients and methods

### Patients

Eighty adolescents of 18 ± 0.4 years old (range 18.1–18.9 years) agreed to undergo semen analysis as part of an andrological screening program conducted in the city of Catania (Southern Italy) in the last 3 years.

The clinical evaluation consisted in the collection of the andrological medical history aimed at assessing the presence of risk factors for male infertility (Table 1) and the physical examination for the assessment of the following parameters: TV; length of the penis in flaccidity; presence of gynecomastia; presence of genital warts; presence of abnormalities of the urethral meatus. They all were in Tanner Stage V.

**Table 1:** Risk factors for infertility found in 80 healthy 18-years-old adolescents

Risk factor	*n*	%
Sexual promiscuity	11	14
Drug abuse	8	10
Alcohol abuse*	15	19
Testicular trauma	5	6
Anabolic–androgenic steroids	3	4

The risk factors not included in this table were considered exclusion criteria

* > 36 g/day

At enrollment the following exclusion criteria were adopted: overweight/obesity [body mass index (BMI) > 25 kg/m^2^], chemotherapy, radiotherapy, endocrine diseases, varicocele (from the first to the third stage according to the Dubin’s classification), cryptorchidism, testicular tumor, cigarette smoke, previous urogenital surgery, urogenital infection in pediatric and adolescent age, orchitis, genetic disorders, use of any medication for other health reasons, presence of sperm antibodies.

### Semen analysis

Semen samples were collected from each adolescent after 4 days of sexual abstinence. The semen analysis was performed following the criteria suggested by the World Health Organization manual [5]. Each specimen was analyzed by two operators for quality control. Semen samples with a leukocyte concentration > 1 million/ml were sent to the microbiology laboratory, and the following microorganisms were searched: *Escherichia coli*, *Enterococcus faecalis*, *Klebsiella*, *Mycoplasma hominis*, *Ureaplasma urealyticum*, *Chlamydia trachomatis*, *Trichomonas vaginalis*, *Gardnerella vaginalis*, *Candida*.

### Seminal leukocyte evaluation (peroxidase test)

The protocol used was adapted from that of Endtz [21]. The working solution was obtained by adding 1 μl of H_2_O_2_ to 20 μl of a 0.09% 3,3′-diaminobenzidine tetrahydrochloride stock solution (DAB, ISOPAC, Sigma, Milan, Italy) in 40% ethanol. 20 μl of semen were incubated with 20 μl of working solution for 5 min at room temperature in each assay. Before setting up the slide, 40 μl of PBS were added. Peroxidase-positive cells were marked by yellow–brown-red staining, while peroxidase-negative cells remained colorless. At least 100 round cells were counted using an optical microscope at 400× magnification and the percentages of peroxidase-positive and -negative cells were evaluated. The total leukocyte count has been expressed in millions per milliliter of semen.

### Evaluation of immature germ cells

Spermatids were differentiated from leukocytes by a semen smear stained using the Papanicolaou technique [22]. Spermatids were identified on the basis of the following parameters: coloration, size, core shape and size (approximately 5 μM), absence of intracellular peroxidase and the absence of leukocyte-specific antigen. Morphologically, multinucleated spermatids were distinguished from polymorphonuclear leukocytes by the presence of a pink color in contrast to the bluish color of polymorphonuclear leukocytes.

### Mixed antiglobulin reaction (MAR) test

All samples were analyzed for the presence of sperm antibodies using the MAR test. The MAR test was performed according with the criteria suggested by the WHO 2010 manual [5]. Briefly, two aliquots of 3.5 µl of semen were prepared and placed on separate microscope slides. One slide was then incubated with 3.5 µl of antibody-positive semen and one with 3.5 µl of antibody-negative semen as control. An aliquot of 3.5 µl of IgG-coated latex particles (beads) was added to each droplet of test and control semen, and properly mixed up by stirring with the pipette tip. An aliquot of 3.5 µl of antiserum against human IgG was added to each semen-bead mixture and mixed-up by stirring with a pipette tip. After covering, the slides were stored horizontally for 3 min at room temperature in a humidified chamber. The methodology was similar for IgA but IgA-coated beads and anti-IgA antibodies were used. The slides were observed at microscope at ×200 magnification after 3 min and again after 10 min, to assess the percentage of motile spermatozoa carrying beads. As the WHO 2010 manual indicates [5], 50% of motile spermatozoa with adherent particles was considered as a threshold value.

### Scrotal ultrasound evaluation

The ultrasound examination was performed with a GX Megas Esaote (Esaote SpA, Genoa, Italy) device, equipped with linear, high-resolution, and high-frequency (7.5 to 14 MHz) probes dedicated to the study of soft body areas, with color Doppler for detecting slow flow and a scanning surface of at least 5 cm. The TV was calculated using the ellipsoid formula (length × width × thickness × 0.52). The testis was considered hypotrophic when it had a volume of less than 12 cm^3^ [23, 24]. TV was evaluated as the mean of the volumes of the right and left testes.

### Statistical analysis

Results are reported as mean ± SD and in percentages throughout the study. The normal distribution of the variables was evaluated with the Shapiro–Wilk test. The association of leukocytospermia with TV and immature germ cells was analyzed using the Pearson correlation or Spearman rank correlation according to the normal distribution of the variables and the results are reported as correlation coefficient (*r*) and *p* value. Data collected from patients with and without leukocytospermia were analyzed by Student’s *t* test. Statistical analysis was performed using SPSS 22.0 for Windows (SPSS Inc., Chicago, IL, USA). A *p* value lower than 0.05 was accepted as statistically significant.

### Ethical approval

This study was carried out at the Division of Andrology and Endocrinology of the University Teaching hospital “G. Rodolico”, University of Catania (Catania, Italy). The protocol was approved by the internal Institutional Review Board and an informed written consent was obtained from each participant after full explanation of the purpose and nature of all procedures used. The study has been conducted in accordance with the principles expressed in the Declaration of Helsinki.

## Results

The medical history showed that 30 out of 80 (38%) adolescent reported promiscuous sexual activity without contraceptive methods, 20/80 (25%) of them referred isolated erectile dysfunction and/or premature ejaculation during sexual activity, 10/80 (12.5%) reported the presence of isolated hypoactive sexual desire. 35/80 (44%) adolescents showed low TV (< 12 ml); 15/80 (30%) low mean penile length in flaccidity using a cut-off of 9.3 cm, equivalent to − 2.5 standard deviations of the normal penile length of an adult man [13] (Table 2).

**Table 2:** Testicular volume (TV) and penile length in 80 healthy 18-years-old adolescents

Parameter	Mean value (± SD)	Percentage of adolescents with abnormal values
Right TV (ml)	17 ± 8	40
Left TV (ml)	17 ± 6	48
Mean TV (ml)	16 ± 7	44
Stretched penile length (cm)	12 ± 6	30

Normal value for TV: ≥ 12 ml; normal value for stretched penile length: ≥ 9.3 cm. Varicocele was an exclusion criterion

Overall, the mean sperm concentration was 40 ± 33 million/ml, the mean value of progressive motility was 30 ± 18%, and the mean value of typical forms was 8 ± 6%. Only 33/80 (41%) boys showed normozoospermia at the semen analysis; 15/80 (19%) isolated oligozoospermia, 12/80 (15%) oligo-asthenozoospermia and 20/80 (25%) oligo-astheno-teratozoospermia.

**Fig. 1:**
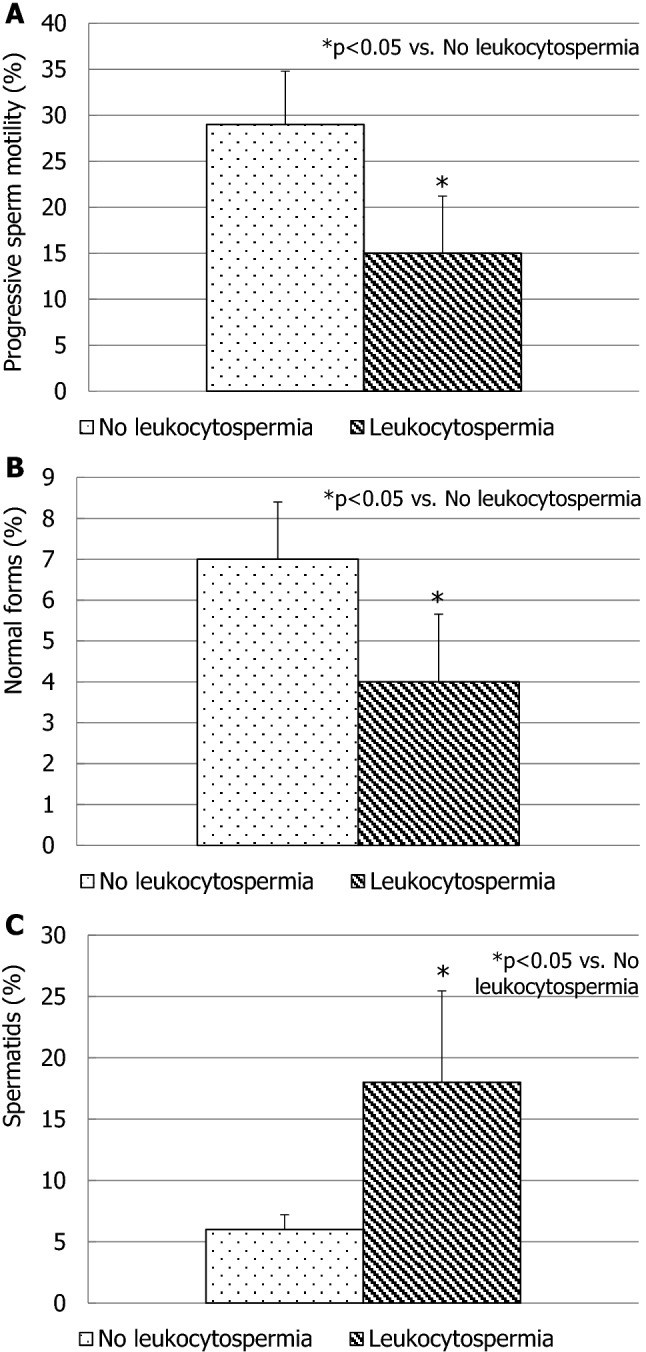
Sperm progressive motility (**a**), morphology (**b**) and spermatid percentage (**c**) in patients with and without leukocytospermia. Standard deviation is indicated by bars

**Fig. 2:**
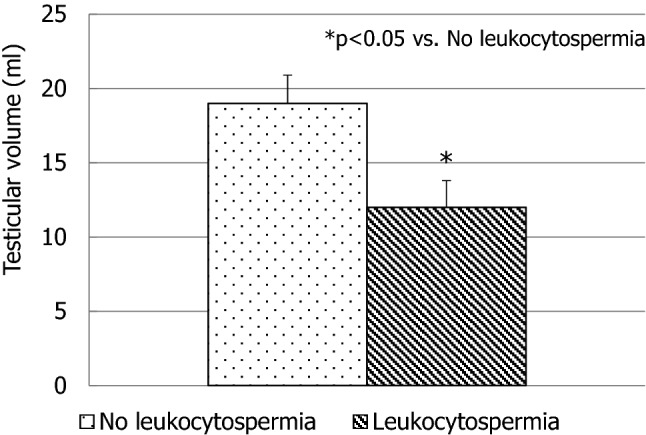
Testicular volume in patients with and without leukocytospermia. Standard deviation is indicated by bars

Notably, 20/80 (25%) adolescents had increased seminal leukocyte concentration (> 1 million/ml). Hence, based on the presence or the absence of leukocytospermia (> 1 million/ml), two groups of patients were identified: those with (2.6 ± 1.1 million/ml) and without (0.6 ± 0.3 million/ml) leukocytospermia (*p* < 0.01). The consequent microbiological assessment revealed the presence of infection in only 5/20 cases. The remaining 15 adolescents with leukocytospermia had no microbial infection in their semen. Progressive sperm motility (Fig. 1, a) and normal forms (Fig. 1, b) resulted significantly lower than those found in adolescents without leukocytospermia. The percentage of spermatids (Fig. 1, c) in the semen was significantly higher. Particularly, the group of patients with leukocytospermia had 16 ± 4 spermatids over 100 spermatozoa, whereas the group without leukocytospermia had 5 ± 2 spermatids over 100 spermatozoa (*p* < 0.01). By contrast, sperm concentration (30 ± 23 million/ml vs. 43 ± 18 million/ml) did not differ significantly in the two groups of adolescents. Finally, adolescents with leukocytospermia had a significantly lower testicular volume (Fig. 2). Testicular hypotrophy was found in 12/20 adolescents with leukocytospermia. No adolescent with testicular hypotrophy was found in the group without leukocytospermia.

The correlation analysis showed a significant inverse relationship between leukocyte concentration and TV (*r* = − 0.83; *p* < 0.05) and the percentage of spermatids (*r* = 0.87; *p* < 0.05).

## Discussion

The results of the present study showed the presence of abnormal sperm parameters in many healthy late adolescents. The alteration of at least one of the main conventional sperm parameters (sperm concentration, progressive motility or morphology) was found in 59% of them. Leukocytospermia was found in 25% of them and resulted idiopathic in the vast majority of them (75%), being the microbiological tests negative for bacterial and fungal infection and the didymo-epididymal ultrasound negative for ultrasound signs of inflammation. In addition, adolescents with leukocytospermia showed a lower sperm progressive motility, percentage of normal forms, TV, and a higher percentage of spermatids compared to adolescents without leukocytospermia. Furthermore, sperm leucocyte concentration correlated negatively with the TV and positively with the percentage of spermatids.

The interpretation of these findings are of great interest because important risk factors for male fertility, such as varicocele, male accessory gland infections, cigarette smoke, obesity, cryptorchidism, testicular tumor, previous urogenital surgery, genetic disorders, or use of any medication for other medical reasons, were taken into account and considered exclusion criteria. Other risk factors or risky lifestyles found in adolescents included in this study, such as drug or alcohol abuse, are listed in Table 1.

The presence of abnormal conventional sperm parameters at such a high frequency shows that primary prevention of male fertility should start very early in life and that the evaluation of testicular growth should be one of the parameters to be taken into consideration. In the event of abnormalities, measurements of serum AMH and inhibin B levels should be undertaken and risky lifestyle identified and adolescent properly counseled to eliminate them to preserve their reproductive health [4, 25].

There is large consensus in the literature for a decline of sperm concentration and total sperm number, which halved in the last forty years without any apparent explanation [26]. This has led to the development of studies that have evaluated sperm parameters in late adolescence, as well as to estimate the prevalence of risky lifestyle for the andrological health in this population, such as smoke, alcohol, drug consumption, unprotected sexual intercourse, which highlights the importance of undergoing to andrological counseling in adolescence. Thus, dysmetabolism and risky lifestyle negatively interfere with male gonadal function at a young age and deserve fully attention [13–16]. Accordingly, recent studies have shown the detrimental impact of lifestyle factors on testicular function in adolescence. A study including 1215 young Danish men aged 18–28 years reported a dose-dependent decline in sperm concentration and total sperm count in marijuana smokers, which accounted for 45% of this cohort or youngsters [13]. In line with these findings, an Italian survey carried out in 10,124 18 years-old male students attending last year of the high school reported a not negligible prevalence of risky lifestyles for the andrological health, such as smoking (32.6%), alcohol intake (80.6%), and use of illegal drugs (46.5%). Unprotected sexual intercourse was reported by 48.3% of these students and of their partners, thus increasing the risk to develop sexual transmitted diseases (STDs). Remarkably, up to 14% had bilateral testicular hypotrophy (TV < 12 ml) at the physical examination, which correlated positively with underweight and heavy alcohol or drug use [14]. Similarly, another Italian study conducted in 360 male high-school students reported an elevated prevalence of cigarette smoke (41.5%), alcohol consumption (9.3%), and cannabis use (19.7%). Their overall awareness about the risk of STDs was low [15]. Finally, a longitudinal study prospectively evaluated the presence of correlations between insulin-resistance and non-alcoholic fatty liver disease (NAFLD) with TV and sperm parameters. Interestingly, this study showed a 50% decrease in sperm output and a TV lower by ⁓20% at the age of 20 in adolescents with NAFLD and insulin-resistance when they were 17 year-old [16].

This evidence supports the findings of the present study. Lifestyles (Table 1) who adolescents were exposed to are potentially able to negatively impact of testicular function. However, the abnormality of mean TV, conventional sperm parameters, and penile length were found in a non-negligible prevalence and the presence of other unknown factors impacting on these outcomes cannot be excluded. Importantly, idiopathic leukocytospermia occurred in 12/80 patients. These adolescents had worse sperm parameters (lower sperm motility and morphology), significantly lower TV and higher spermatid concentration compared to adolescents without leukocytospermia. This group of adolescent may be at risk of a testicular damage during youth, which may predispose them to a decreased sperm output later in life. Furthermore, to our knowledge, this is the first report of idiopathic (amicrobial) leukocytospermia in adolescents and, therefore, its interpretation/significance is not fully codified.

Leukocytospermia is an established marker of inflammation and it has been associated with increased semen levels of interleukin-6 and tumor necrosis factor-α, both inflammatory cytokines, in infertile patients [27]. Leukocytes are also involved in the pathogenesis of autoimmune inflammation of seminal ducts [28]. The blood-testis barrier (BTB) is made by Sertoli cells to defend auto-immunogenic spermatids from the self-immune system attack. However, some penetrating lymphocytes lie very close to the germ cells, being retrieved in mice tubuli recti, rete testis and epididymis, but not in the seminiferous tubules, under normal conditions. These cells have been advocated to the pathogenesis of autoimmune inflammation of tubuli recti, rete testis and epididymis [28]. The disruption of BTB integrity is involved in the pathogenesis of worse seminal phenotypes, such as Sertoli cell only syndrome or hypospermatogenesis [29]. The BTB, which makes the testis an immunologically privileged organ, literary divides the epithelium of seminiferous tubules into two compartments: the basal one, including germ cell in I/II meiosis and the adluminal one, where post-meiotic spermatids and spermatozoa can be retrieved [30]. The increase in immature germ cells in the semen, including spermatocytes and spermatids, is indicative for testicular damage, as the WHO manual indicates [5]. The higher leukocyte and spermatid percentage reported in the adolescents included in the present study may indicate the presence of a testicular tubulopathy, which is strongly supported by the presence of a decreased TV. TV represents a widely accepted marker of testicular function since it correlates positively with conventional sperm parameters [31]. While in the pre-pubertal phase TV reflects Sertoli cell proliferation, it is related with the amount of spermatogenesis in testicular tubules during the post-pubertal phase [2]. Hence, the concomitant presence of a reduced TV supports the hypothesis of tubular dysfunction. However, longitudinal studies are warranted to confirm this hypothesis.

We are aware of some important study limitations. First of all, the cross-sectional nature of the study does not allow any conclusive data regarding these young boys and their possibility to recover sperm parameters naturally. Second, we did not perform HPV analysis as a possible cause of leukocytospermia and we are aware that its incidence amongst young boys who have unprotected sex is quite high. In addition, sperm culture was the only microbiological test which patients underwent to. This may have underestimated the real prevalence of bacterial infection. Last but not least, the impossibility to investigate any organic cause of erectile dysfunction and correlation with sexual hormones as well as with penile duplex ultrasound [31].

## Conclusion

In conclusion, we have found the presence of alteration of at least one of the conventional sperm parameters in more than a half of healthy adolescent men. Furthermore, a distinct phenotype characterized by leukocytospermia, increased spermatid concentration and low TV was identified, possibly indicating the presence of testicular tubulopathy. We have also found a non-negligible prevalence of testicular hypotrophy and low penile growth. In line with previous findings, our data strongly highlight the importance of primary prevention of male infertility, which should be accomplished starting from pre-pubertal age by monitoring testicular growth, measuring AMH and inhibin B serum levels in selected cases^31^ and counselling boys and adolescents for identifying/eliminating risky lifestyles for andrological health.
